# Supplementary material for: Identifying putative substrates of Calpain-15 in neurodevelopment
Source: PLoS One. 2025 Apr 16;20(4):e0319489. doi: 10.1371/journal.pone.0319489 (PMC12002525; doi:10.1371/journal.pone.0319489)
Supplement: S1 Supplemental Data — (PDF) [file pone.0319489.s001.pdf]

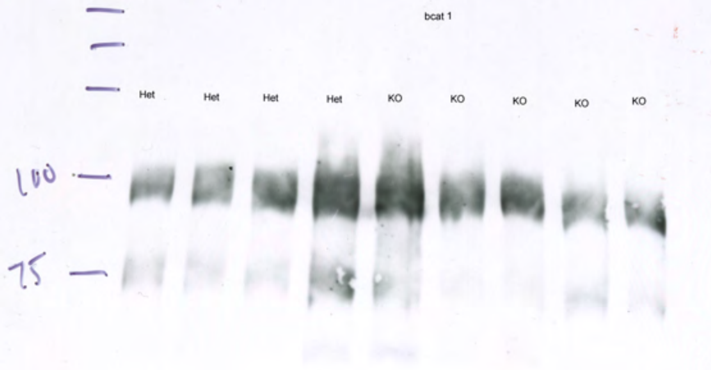
bcat 1
Het
Het
Het
Het
KO
KO
KO
KO
KO
100
75

bcat1

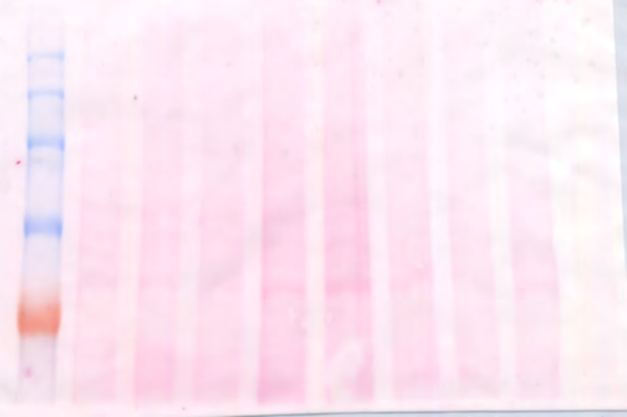

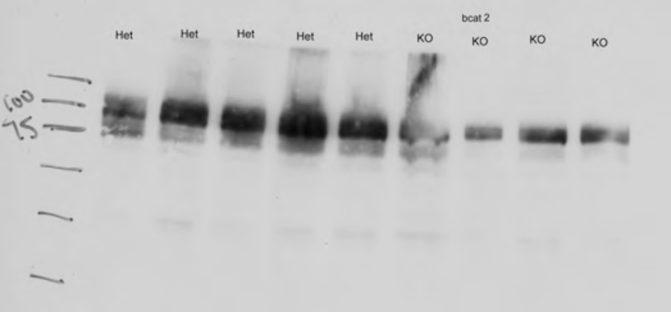
bcat 2
Het
Het
Het
Het
Het
KO
KO
KO
KO
100
75

bcat2

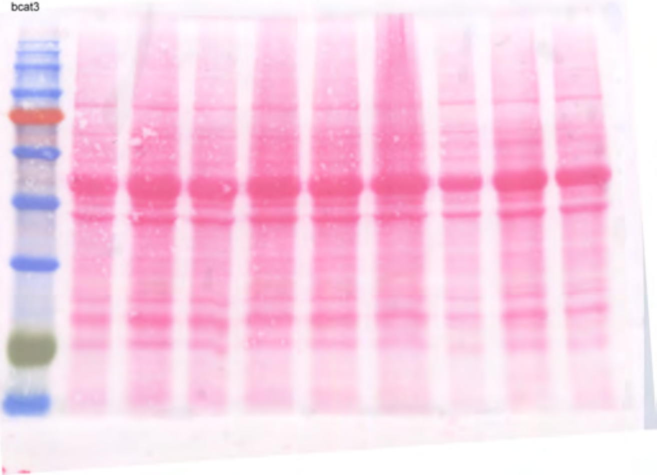

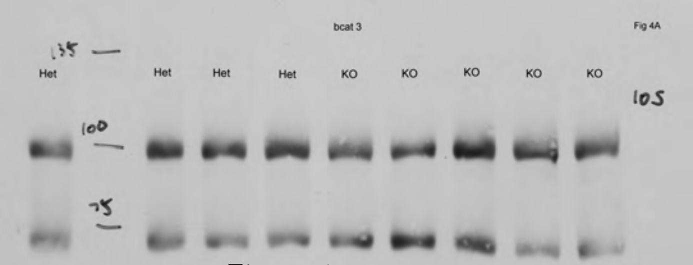

--Figure 4a--------

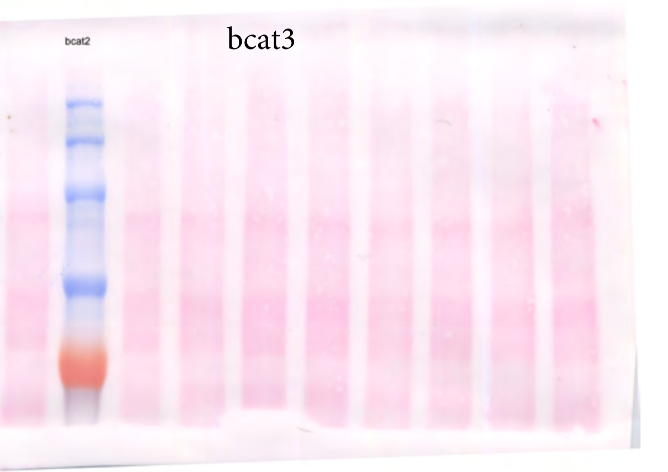
bcat2
bcat3

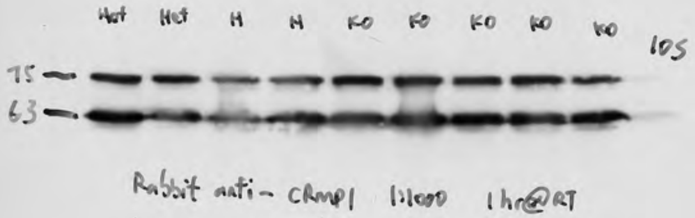
Het Het H H KO KO KO KO KO
10S
75
63
Rabbit anti- CRMP1 1:1000 1hr@RT

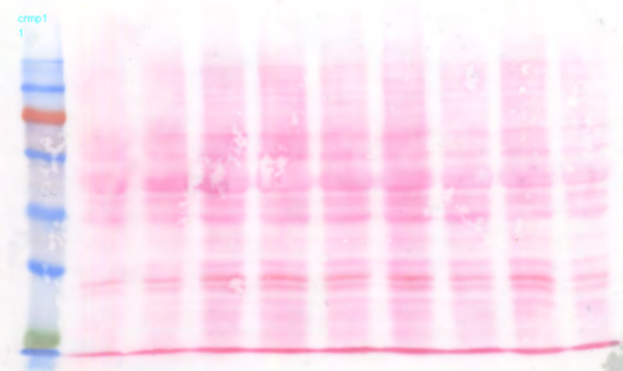
crmp1
1

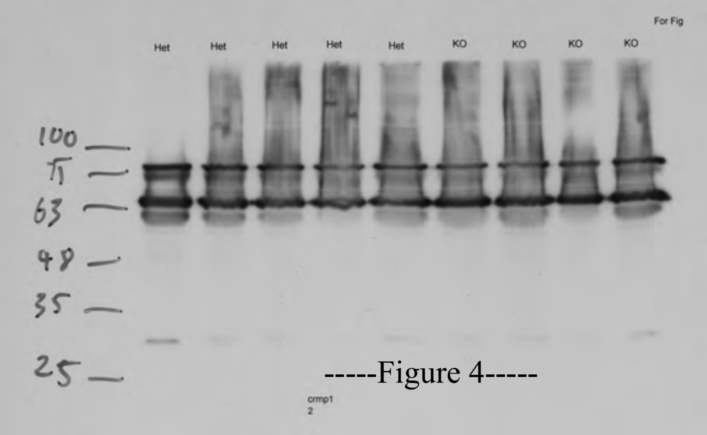

-----Figure 4-----

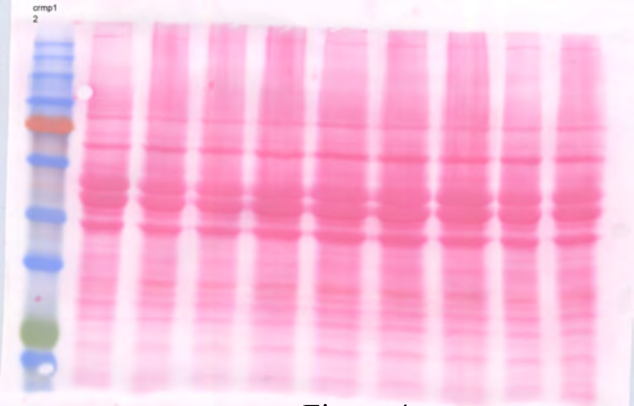

-----Figure 4-----

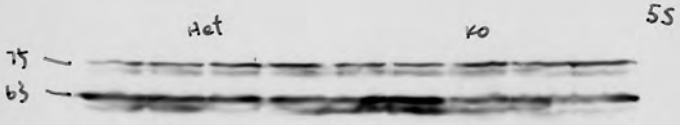

Rabbit anti-CRMP1 1:1250 1hr @ RT

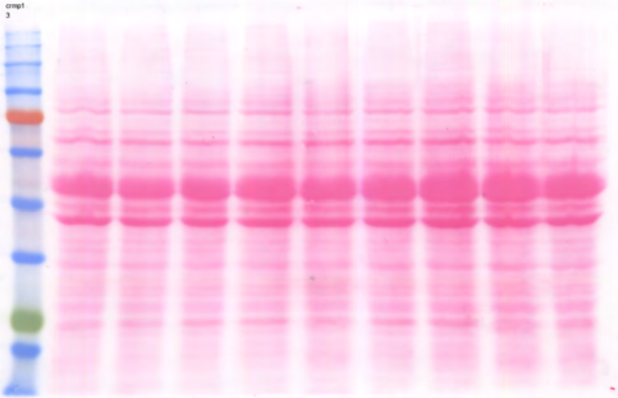
crmp1
3

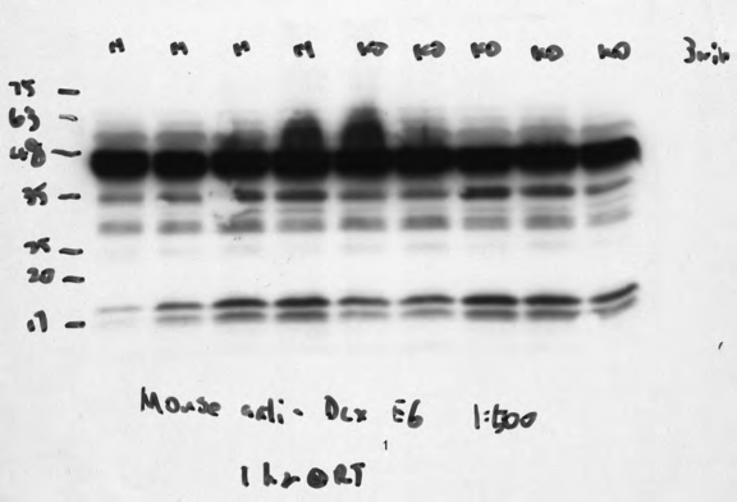
M
M
M
M
KO
KO
KO
KO
KO
75
63
48
35
25
20
17
Mouse anti-Dcx E6 1:500
1 hr @ RT

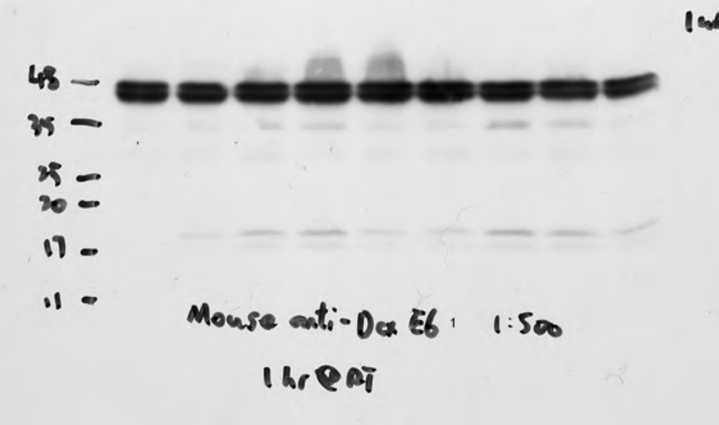
48
35
25
20
17
11
Mouse anti-Dcx E6 1:500
1hr@RT

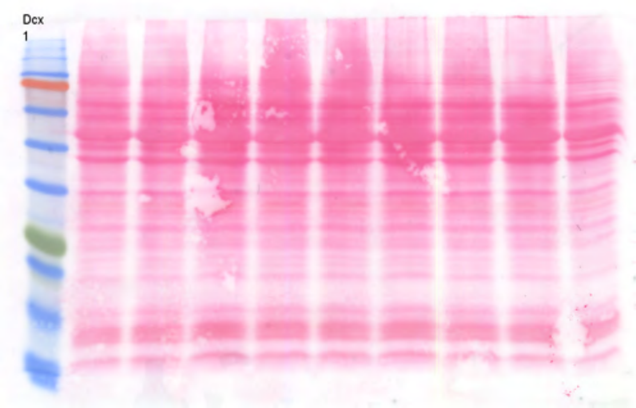
Dcx
1

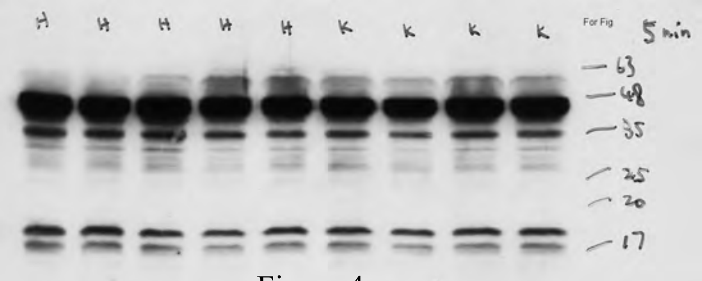

-----Figure 4-----

Mouse anti-Dcx E6 1:500 1hr @RT

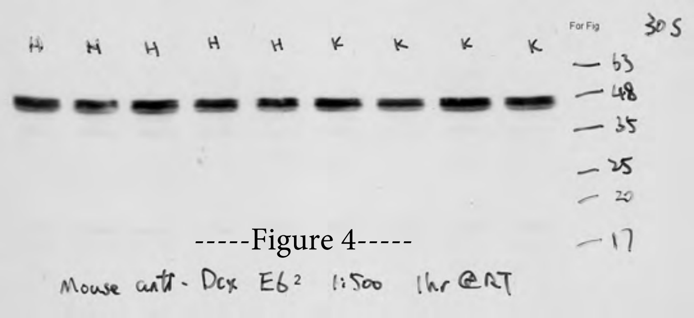
For Fig
30 S
H H H H H K K K K
63
48
35
25
20
17
Mouse anti - Dcx E6 2 1:500 1hr @RT

-----Figure 4-----

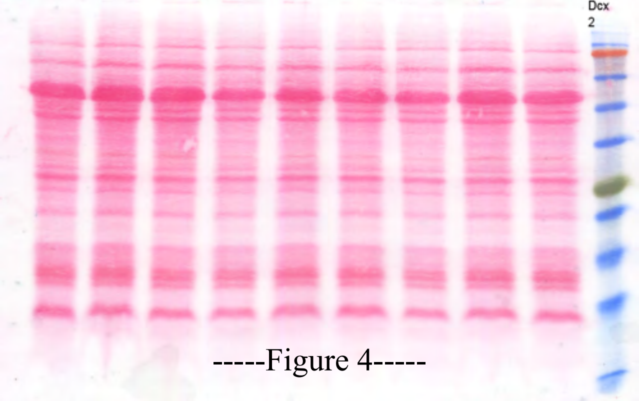

-----Figure 4-----

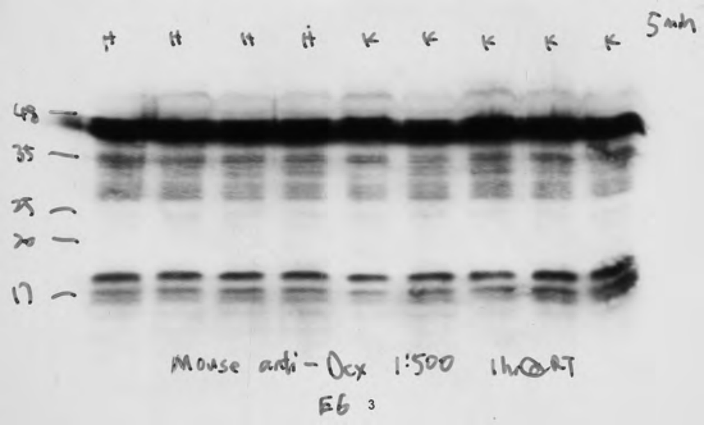
H H H H K K K K K
5 min
48
35
25
20
17
Mouse anti-Dcx 1:500 1hr@RT
E6 3

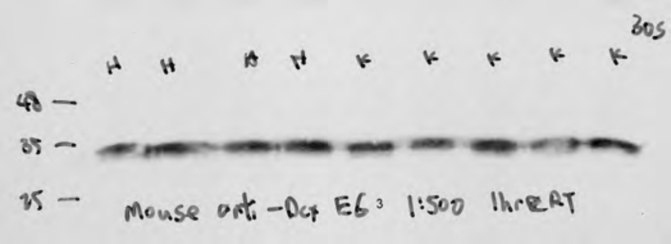
305
H H H H K K K K K
48 —
35 —
25 —
Mouse anti-Dcx E6 3 1:500 1hr@RT

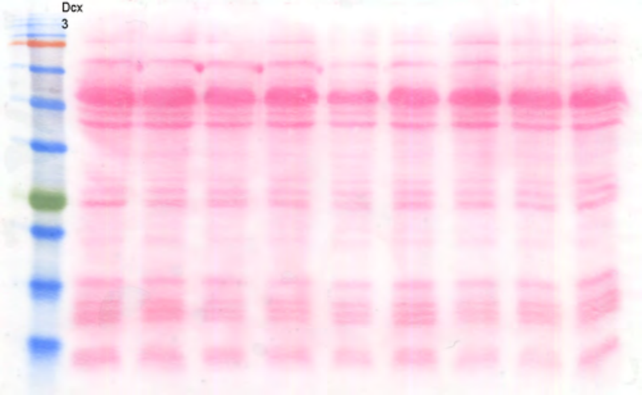
Dcx
3

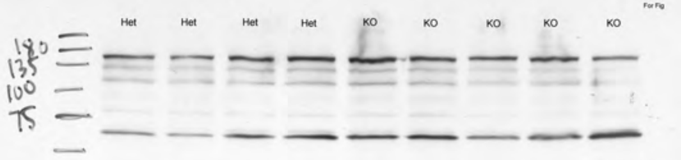

-----Figure 2 -----

Dhx9 1

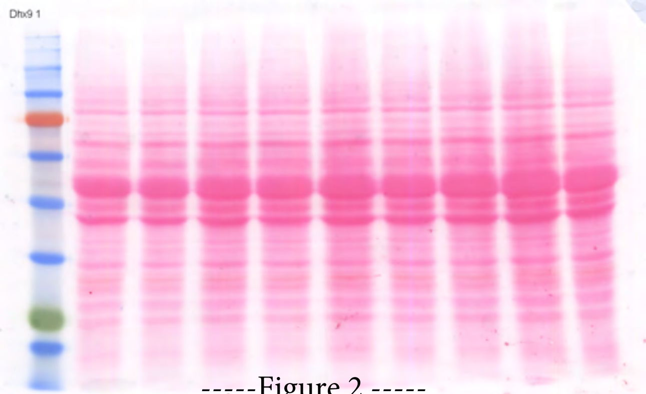

-----Figure 2 -----

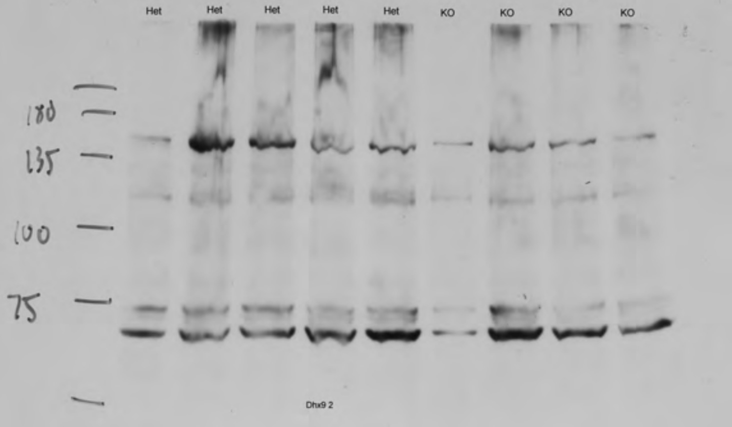

Het
Het
Het
Het
Het
KO
KO
KO
KO
180
135
100
75
Dhx9 2

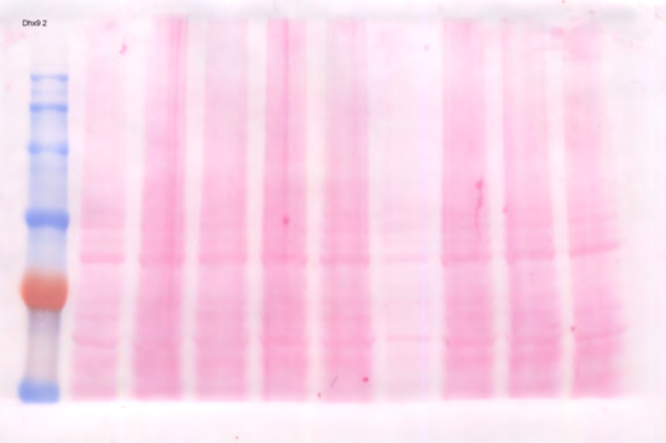
Dhx9 2

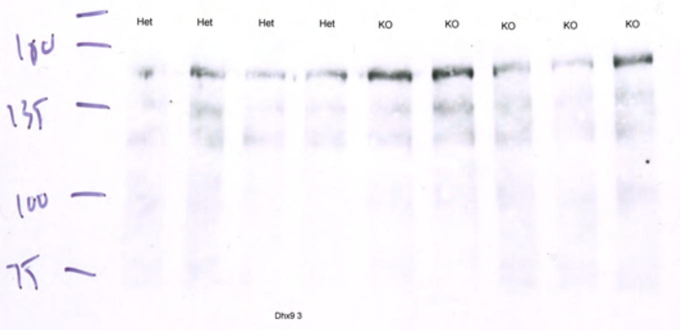

180
135
100
75
Het
Het
Het
Het
KO
KO
KO
KO
KO
Dhx9 3

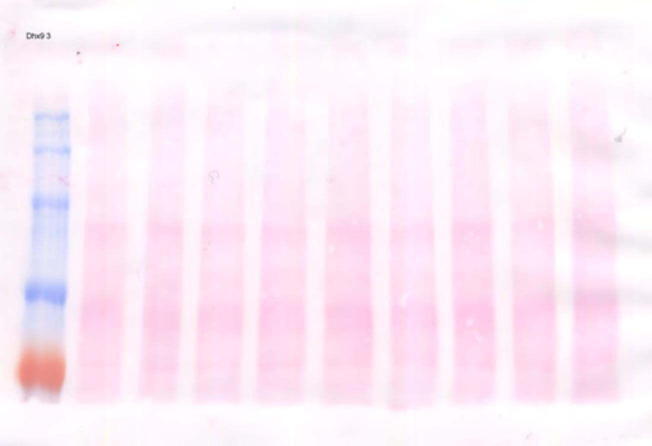
Dhx9 3

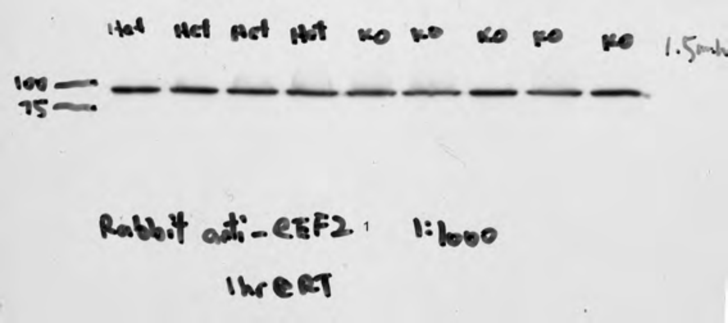
Het Het Het Het KO KO KO KO KO
1.5min
100
75
Rabbit anti-eEF2 1:1000
1hr @ RT

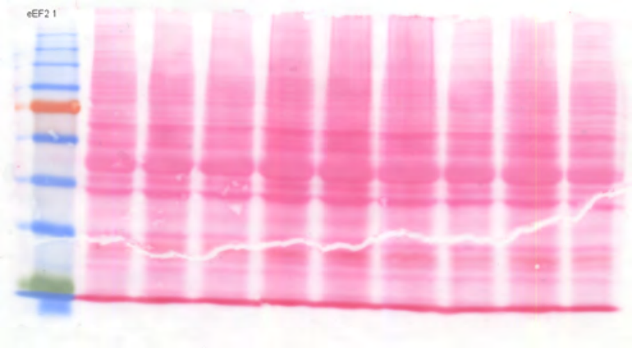
eEF2 1

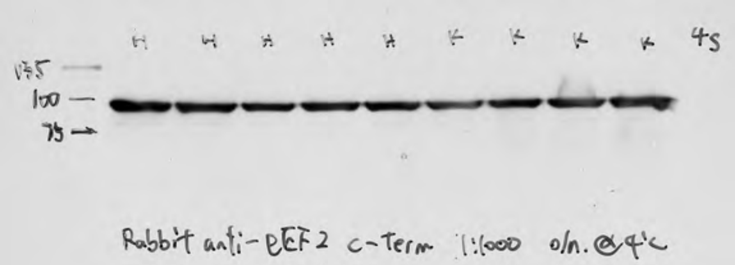
H H H H H K K K K 4S
135
100
75
Rabbit anti-eEF2 c-Term 1:1000 o/n. @4°C

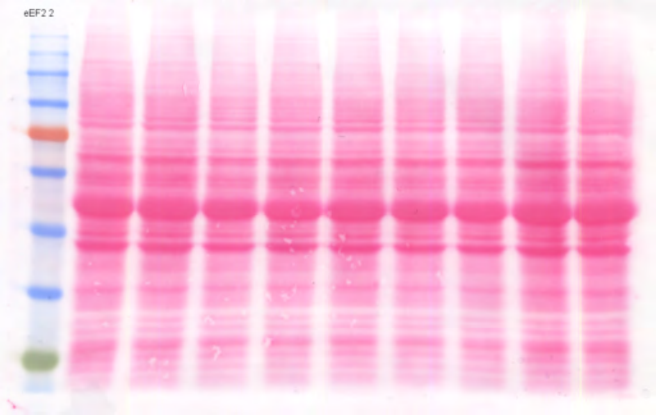
eEF2 2

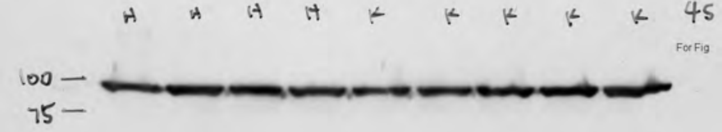

------Figure 4------

Rabbit anti-eEF2 C-term 1:1000 o/n. @4°C

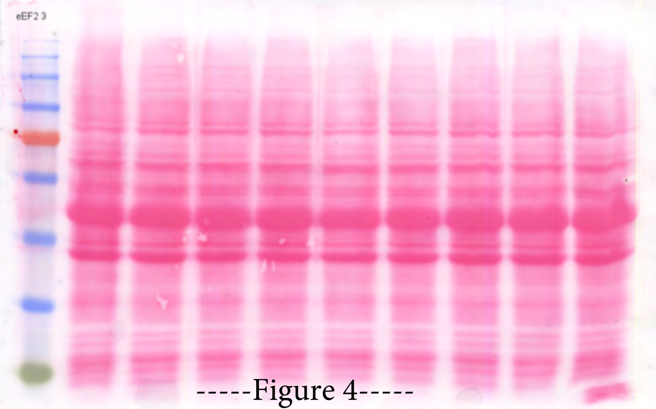

-----Figure 4-----

-----Figure 1A------------

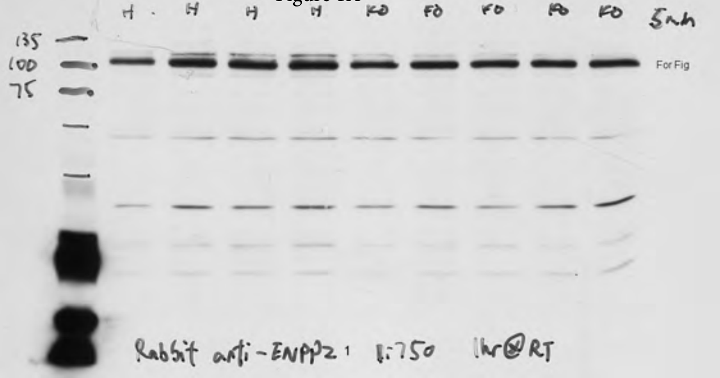

------ Figure 1------

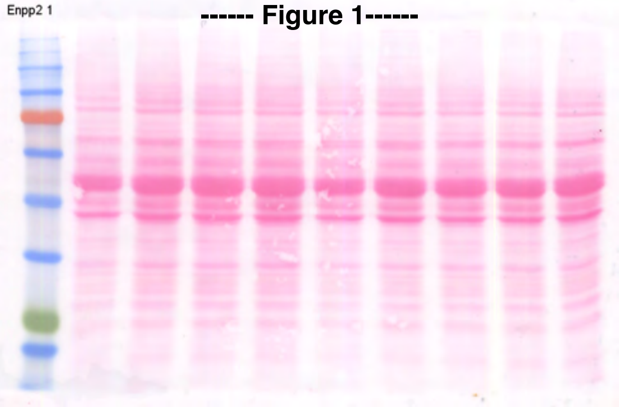

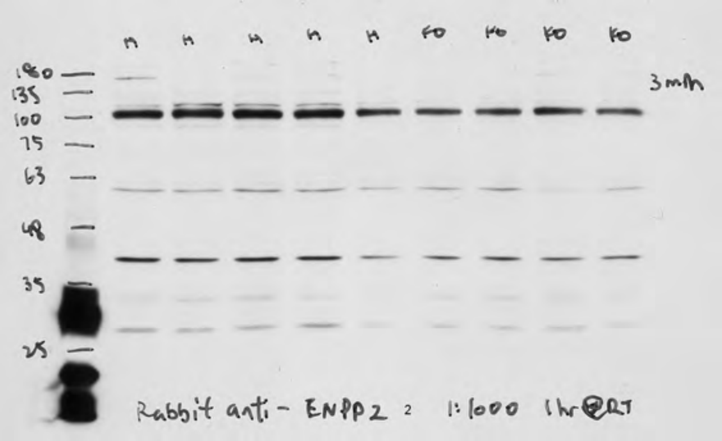
H
H
H
H
H
KO
KO
KO
KO
180
135
100
75
63
48
35
25
3min
Rabbit anti-ENPP2 1:1000 1hr@RT

Enpp2 2

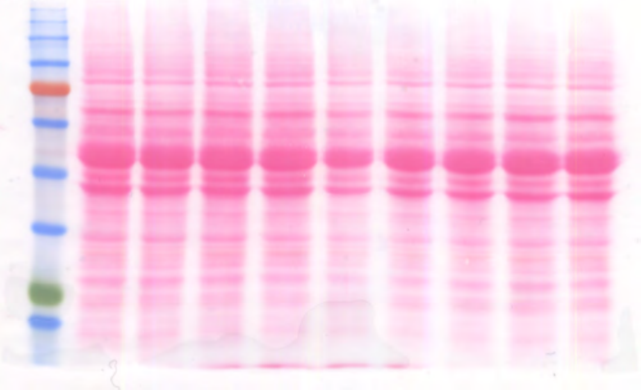

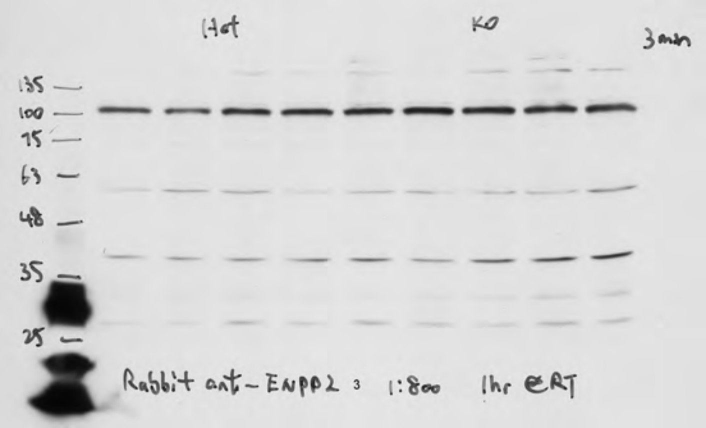
Het
KO
3 min
135
100
75
63
48
35
25
Rabbit anti-ENPP2 3 1:800 1hr @RT

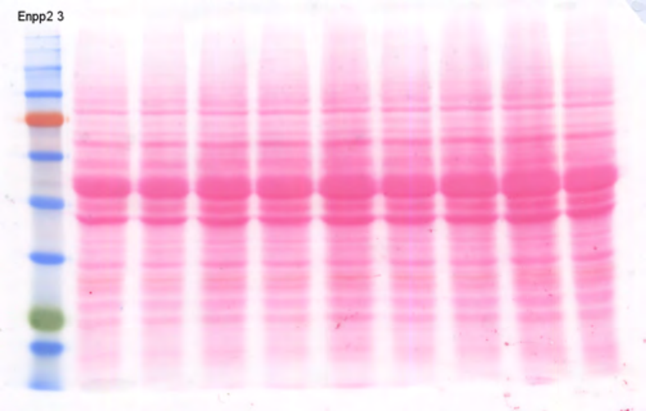
Enpp2 3

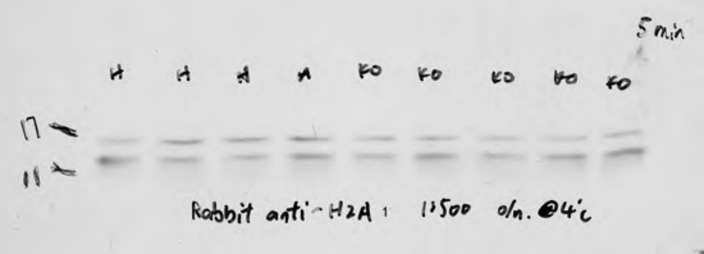
5 min
H H H H KO KO KO KO KO
17
11
Rabbit anti-H2A 1 1:500 o/n. @4°c

H2A 1

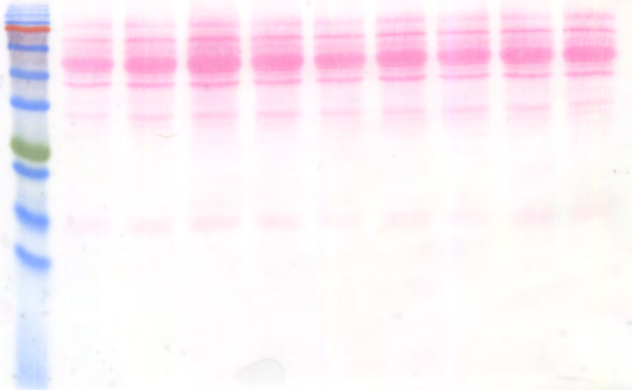

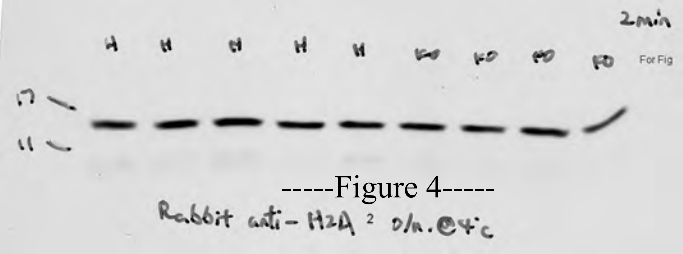

-----Figure 4-----

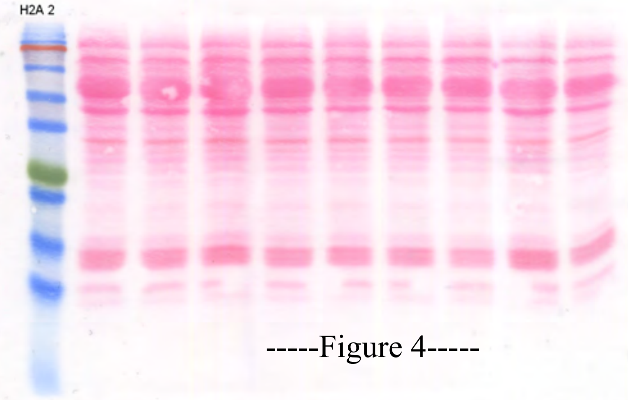

-----Figure 4-----

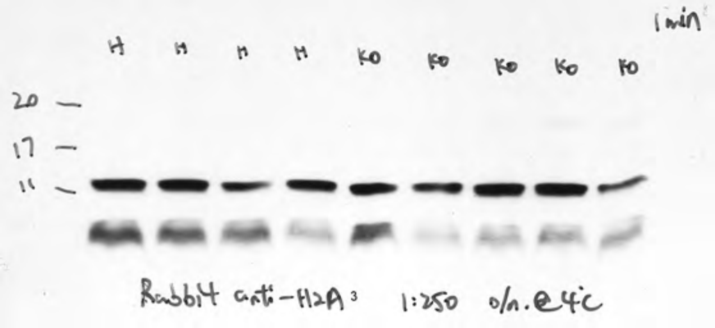
1 min
H
H
H
H
KO
KO
KO
KO
KO
20 —
17 —
11 —
Rabbit anti-H2A3 1:250 o/n.@4C

H2A 3

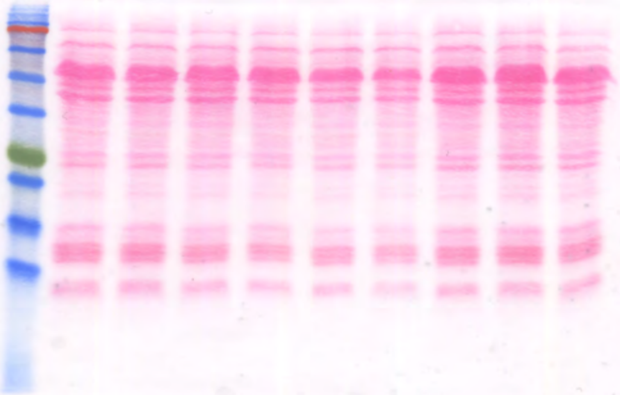

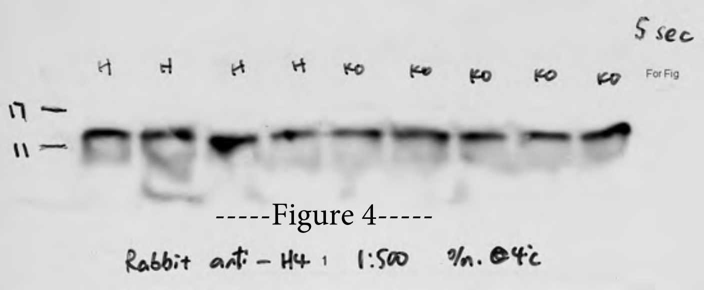

-----Figure 4-----

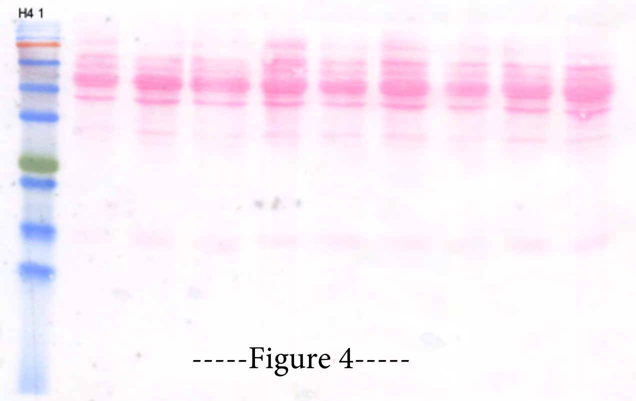

-----Figure 4-----

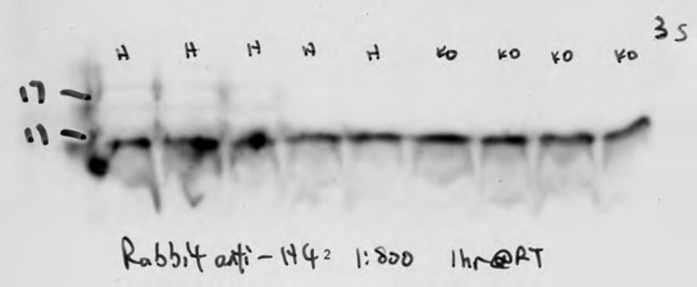
3s
H H H H H KO KO KO KO
17
11
Rabbit anti-H4 2 1:800 1hr@RT

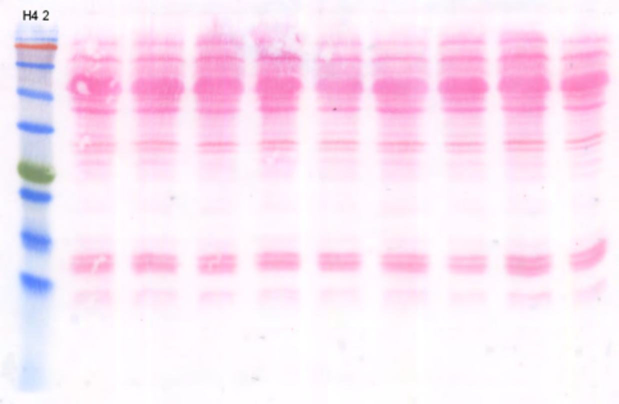
H4 2

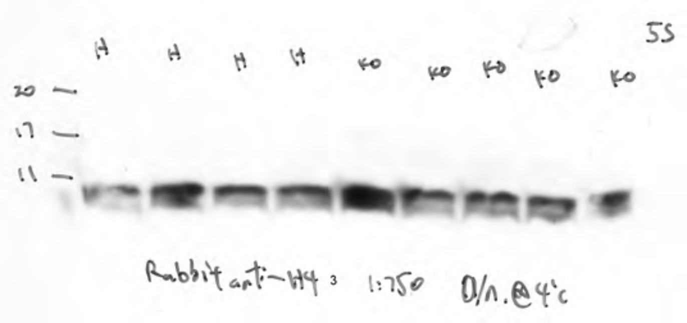
5S
H H H H KO KO KO KO KO
20
17
11
Rabbit anti-H4 1:750 O/N @ 4°C

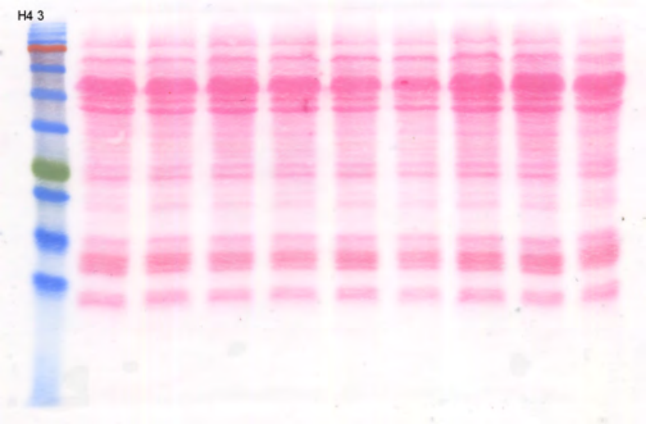
H4 3

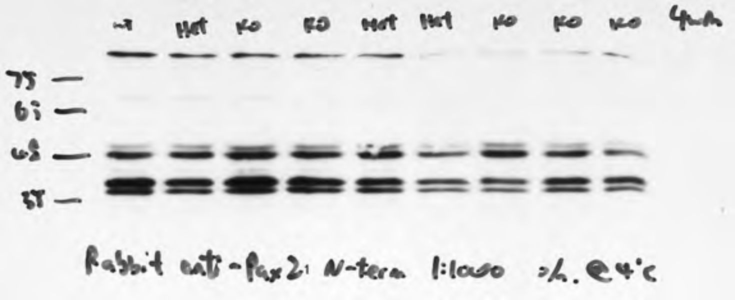
WT Het KO KO Het Het KO KO KO 4mth
75
63
48
35
Rabbit anti-Pax2 N-term 1:1000 o/n @4°C

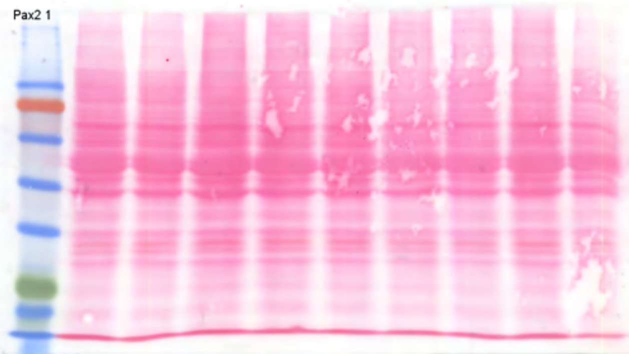
Pax2 1

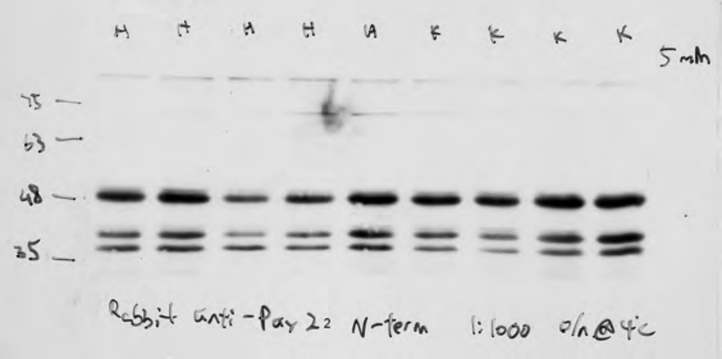
H H H H H K K K K
5 min
75
63
48
35
Rabbit anti-Pax 22 N-term 1:1000 o/n @ 4°C

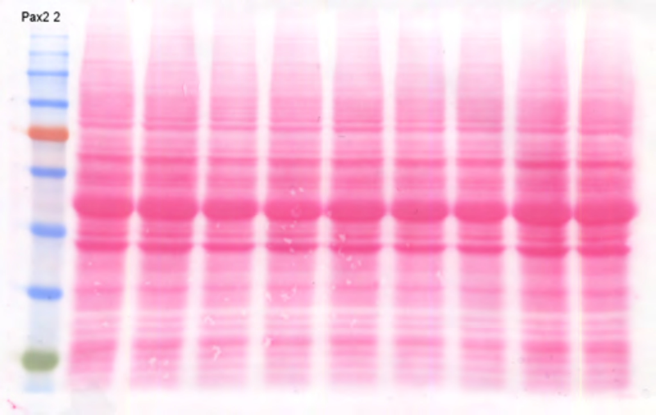
Pax2 2

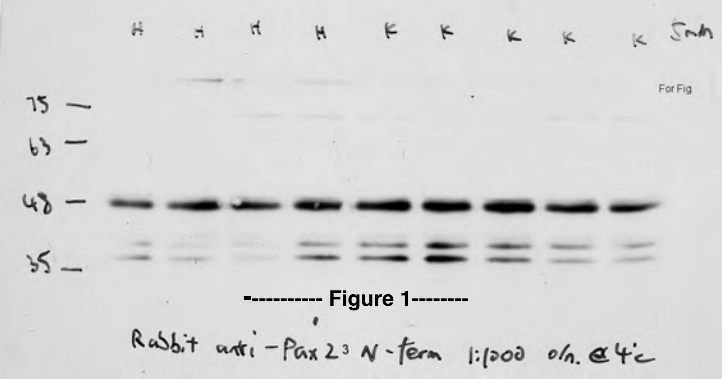
H
H
H
H
K
K
K
K
K
For Fig
75 —
63 —
48 —
35 —
Rabbit anti-Pax2 N-term 1:1000 o/n. @4°c

-----------Figure 1--------

--------- Figure 1----------

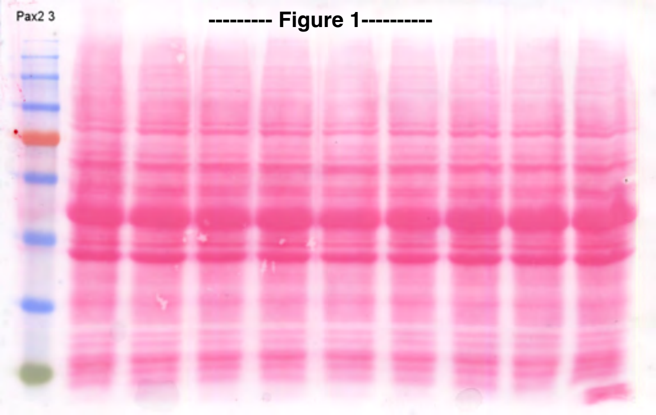

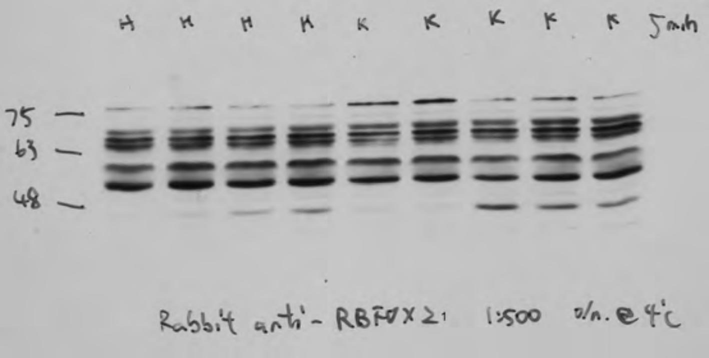
H H H H K K K K K 5min
75
63
48
Rabbit anti- RBFOX2 1:500 o/n. @ 4°C

Rbfox2 1

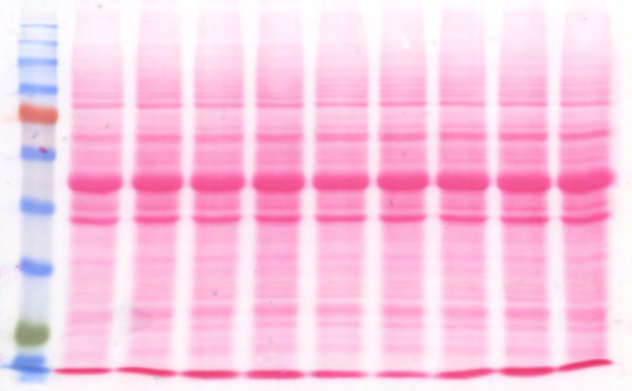

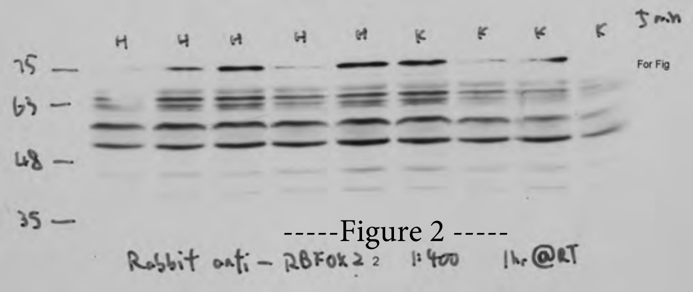
5 min
H H H H H K K K K
75
63
48
35
For Fig
Rabbit anti - RBFOX2 2 1:400 1hr @RT

-----Figure 2 -----

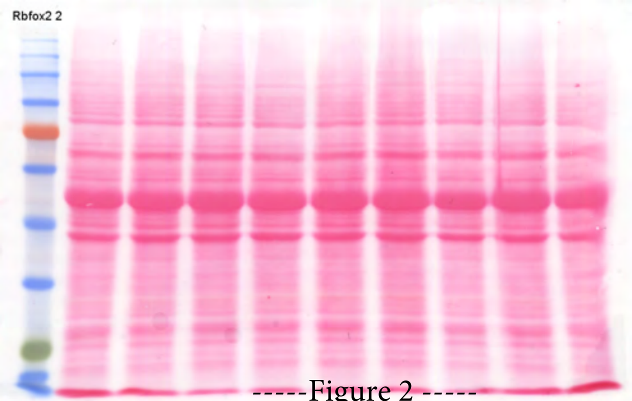

-----Figure 2 -----

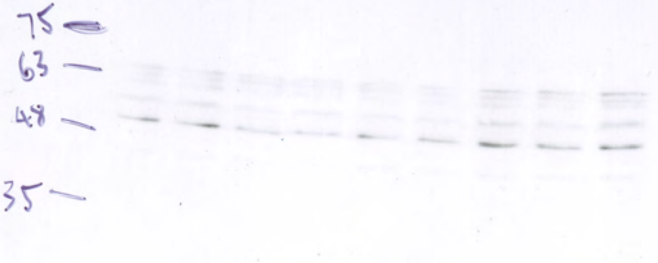

Rbfox2 3

Rbfox2 3

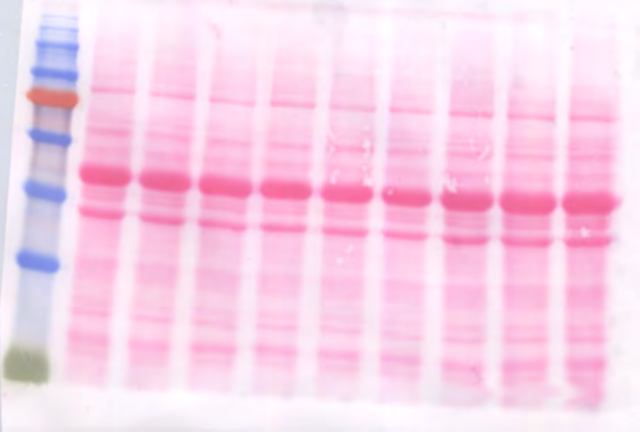

For Fig

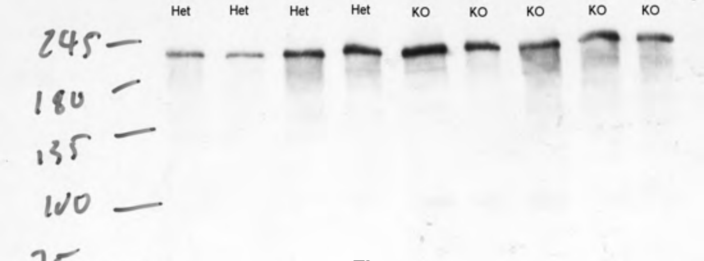

---------Figure 1--------

Smarca4 1

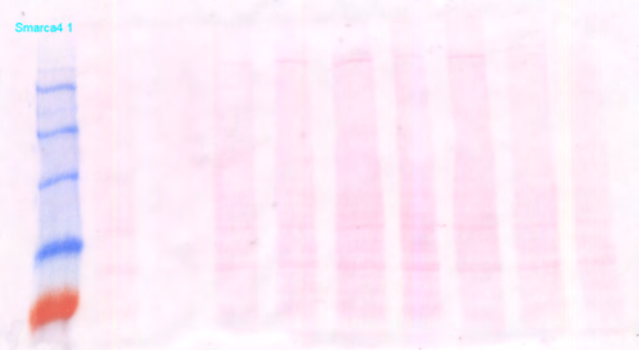

--------Figure 1--------

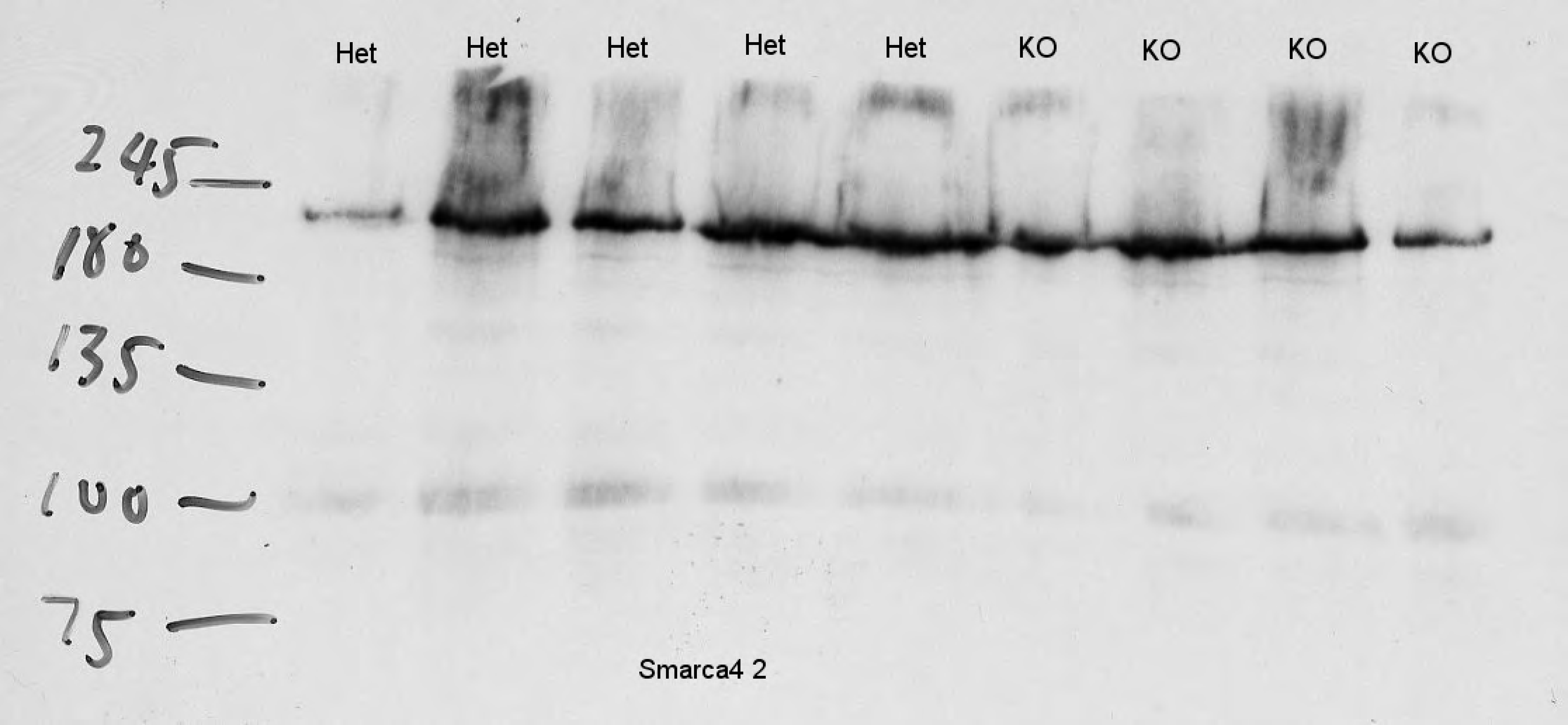
Het
Het
Het
Het
Het
KO
KO
KO
KO
245
180
135
100
75
Smarca4 2

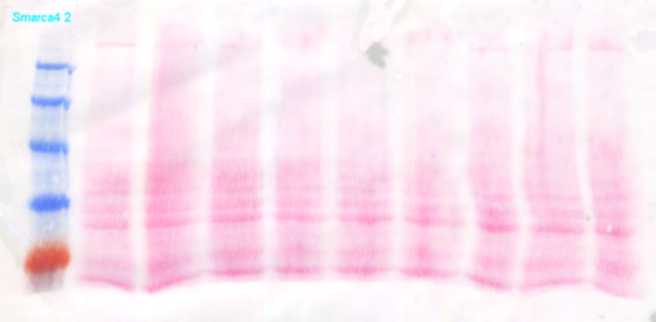
Smarca4 2

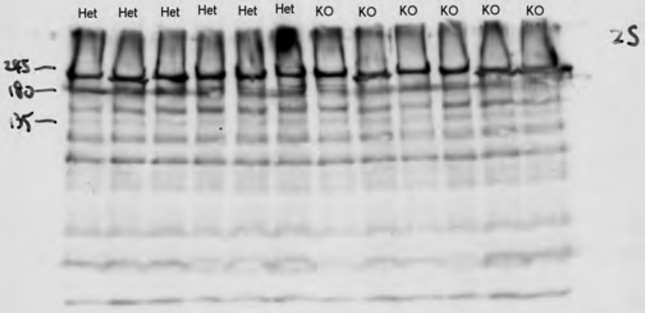

Rabbit anti-Smarca4 3 1:1000 1hr@RT

Smarca4 3

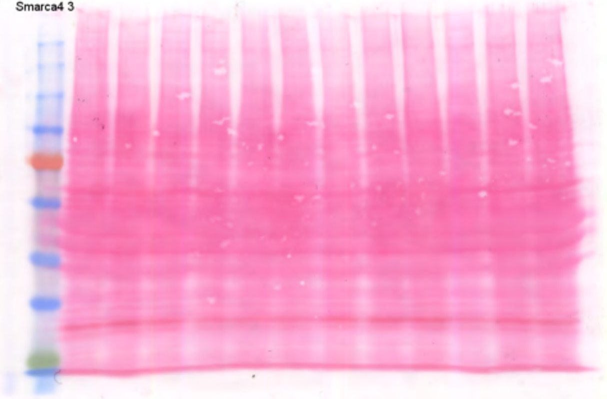

------Figure 4------

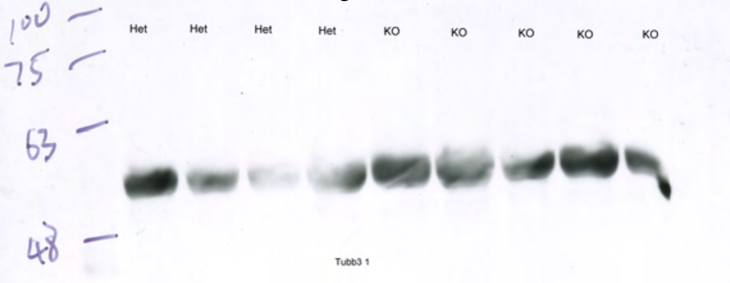

-----Figure 4-----

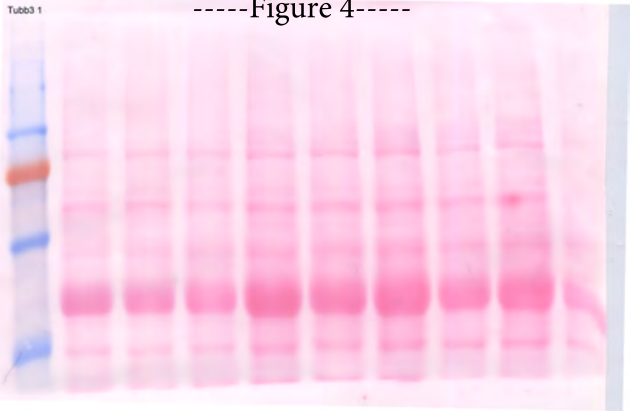

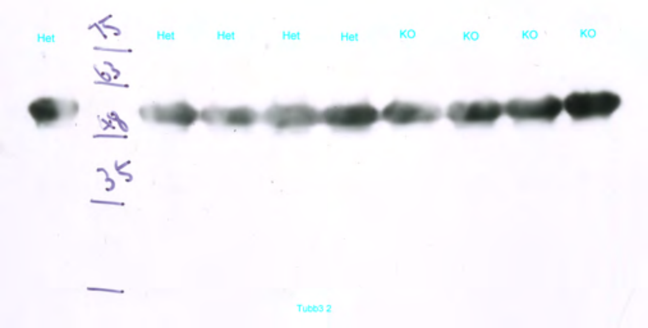
Het
75
Het
Het
Het
Het
KO
KO
KO
KO
63
48
35
Tubb3 2

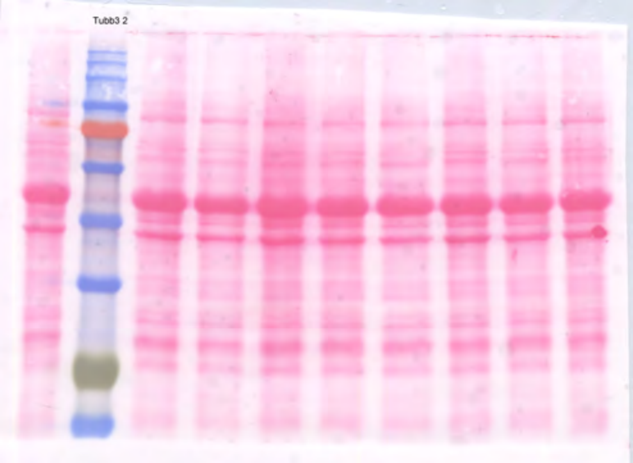
Tubb3 2

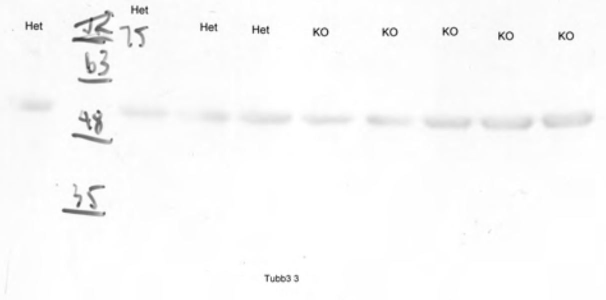
Het
Het
75
63
48
35
Het
Het
KO
KO
KO
KO
KO
Tubb3 3

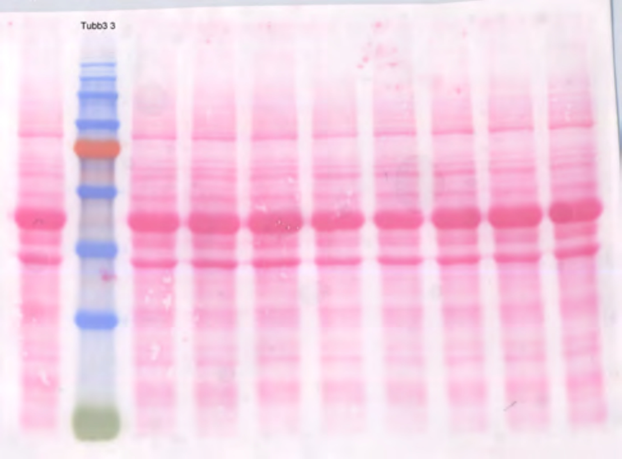
Tubb3 3

------Figure 1-----

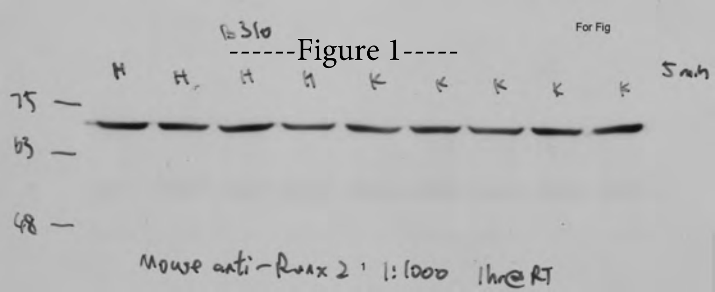

------Figure 1-----

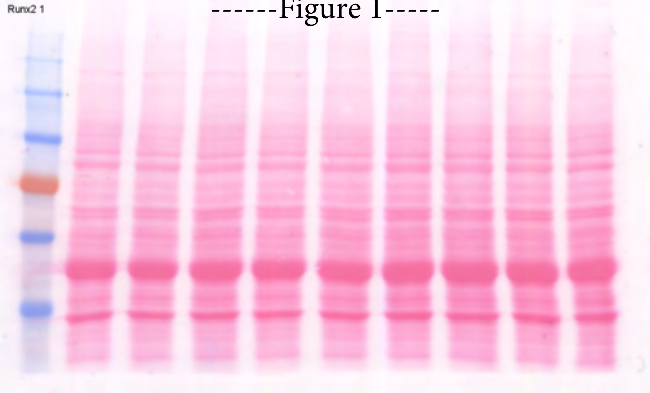

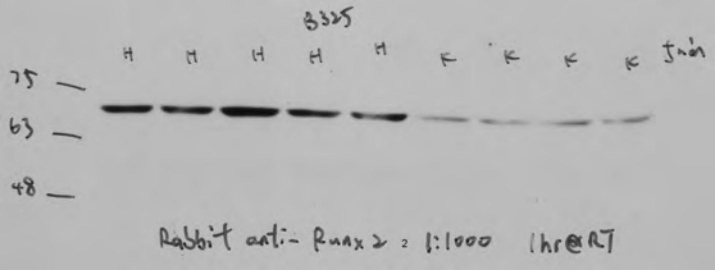
B325
H H H H H K K K K
75
63
48
Rabbit anti-Runx2 1:1000 1hr@RT

Runx2 2

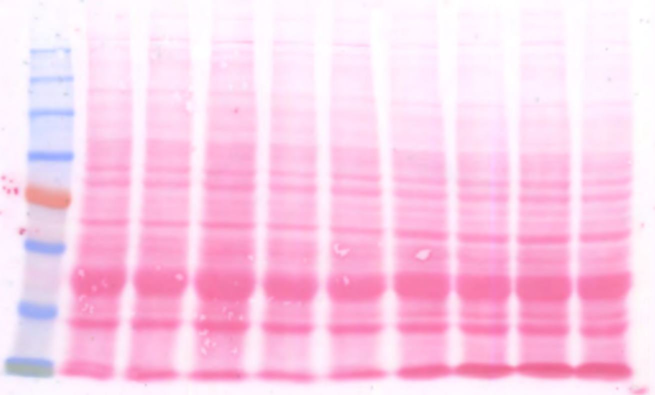

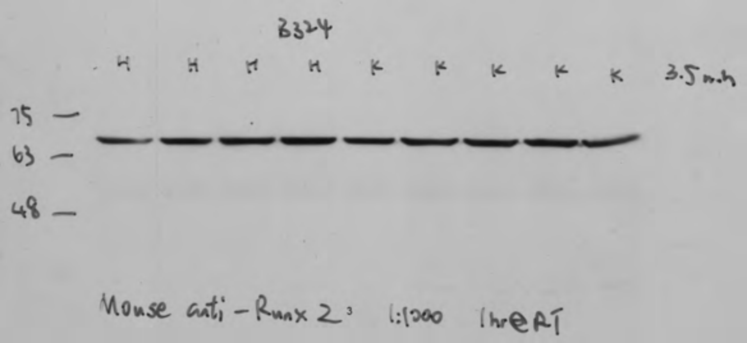
B324
H H H H K K K K K
3.5 min
75
63
48
Mouse anti-Runx 2 1:1000 1hr@RT

Runx2 3

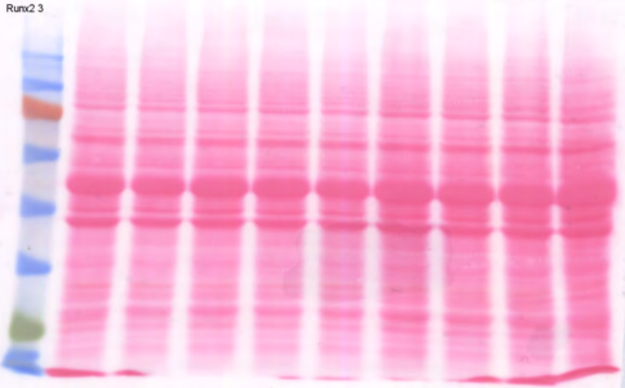

------Figure 1-----

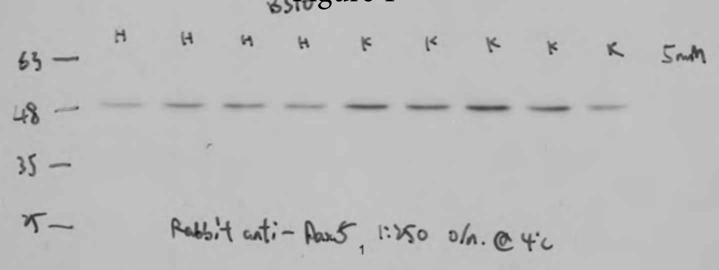

------Figure 1-----

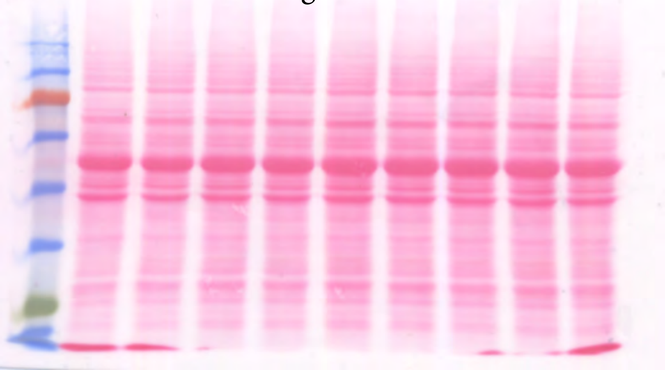

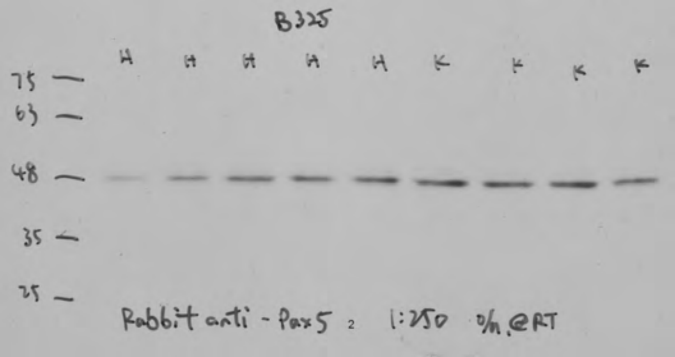
B325
H
H
H
H
H
K
K
K
K
75
63
48
35
25
Rabbit anti-Pax5 2 1:250 o/n @RT

Pax5 2

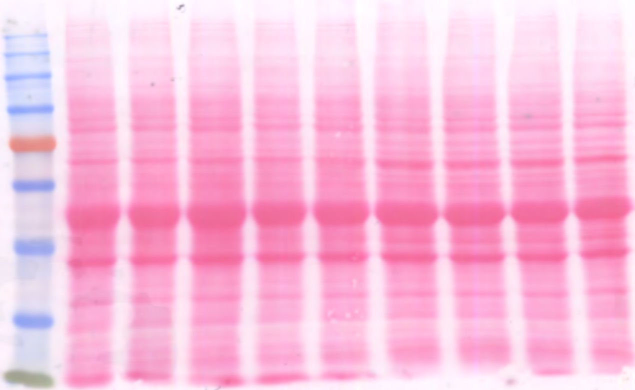

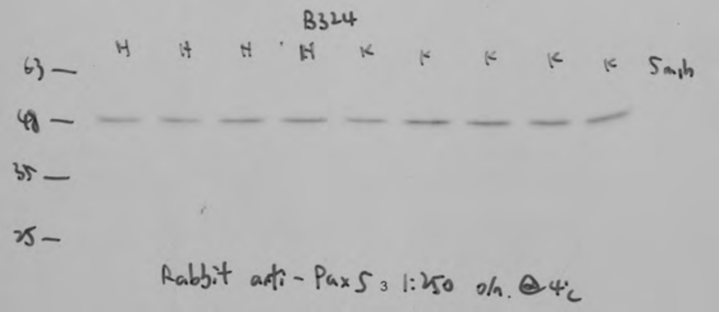
B324
H H H H K K K K K 5min
63 —
48 —
35 —
25 —
Rabbit anti-Pax5 3 1:250 o/n. @ 4°C

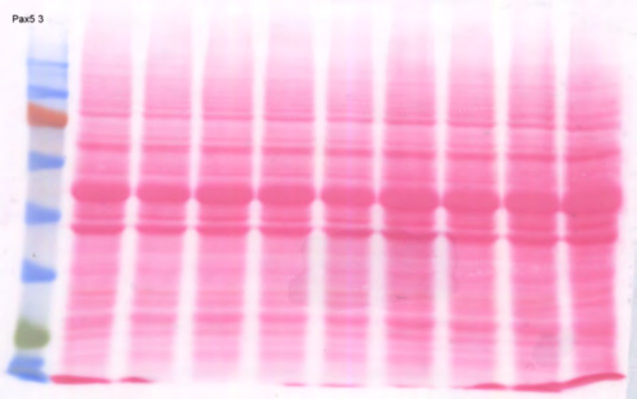
Pax5 3
