# Supplementary material for: Identifying putative substrates of Calpain-15 in neurodevelopment
Source: PLoS One. 2025 Apr 16;20(4):e0319489. doi: 10.1371/journal.pone.0319489 (PMC12002525; doi:10.1371/journal.pone.0319489)
Supplement: Attachment — Submitted filename: response to reviews_ final.pdf [file pone.0319489.s008.pdf]

Dear Editor,

Thank you for the constructive comments on our manuscript. All reviewers suggested useful changes and additional information to be included in our manuscript. Therefore, we performed a thorough re-write and have addressed all the points raised by the reviewers to the best of our ability.   Below is a detailed rebuttal letter to the academic editor and reviewers. We hope this satisfies PLOS One's publication criteria.

Reviewer #1.

Thank you for your positive comments. We have fixed the typographical errors pointed out by the reviewer.

Reviewer #2

The authors stated using both sexes of CapN15 KO mice. While it might be difficult to correctly identify the mice sex at P2, does the author have any evidence that there are no sex differences in these KO mice and if that influences the 38 mRNAs found to have significant differential gene expression changes? Please address this possibility.

(additional editor) *Include validation that the different sex of the mice did not affect the results*

For the RNA-SEQ experiments, the differential gene expression analysis considered the sex of the animal as the RNA-SEQ data for each sample can easily detect the sex of the animal. Thus, sex of the animal did not influence the mRNAs found to have expression changes.

Line 227-229 (clean version) "When **sex** and litter were included as variables, there were 38 mRNAs with significant changes in abundance with a differential gene expression (DGE) analysis and FDR cut off of 0.05 (Fig. 1A)."

Previous studies on these mice did not detect sex differences in the effects of the loss of Capn 15 (Zha et al, 2020 Hum. Mol. Gen; Zha et al, 2021 Neuroscience; Zha et al 2023 Behavioral Brain Res).

2. Figure 1A and 1B: It is definitely critical to represent the up- and down-regulated genes in a figure rather than a table since it is being shown as a figure. The current version can be included as a table by itself.

Agreed and great suggestion. We have replaced the table (Fig. 1A) with a volcano plot and have taken Fig. 1A and B and now present them as tables (new Table 1 and Table 2) as suggested.

3. The authors tested only two of the hits from the LISA analysis screen: Pax and Smarca4. Pax2 was shown to be significantly increased. I suggest the authors test and validate more than those two.

We have tested two additional hits from the LISA screen as suggested: PAX5, similar to PAX2 showed a significant increase in levels, while RUNX2 did not show a difference.

Furthermore, additional work such as investigating effect of Pax2 inhibition can be performed. The same can be performed for Dcx that was suggested to be a putative cleavage product of Capn15.

We agree that the next step in this research would be functional experiments to test the effects of increasing levels of these transcription factors on neurodevelopment. However, these experiments would take additional years of research and are beyond the scope of the present manuscript. Similarly, functional experiments on knock-in mice that remove the putative cleavage site of Dcx by Capn15 is also beyond the scope of the present manuscript. We have included discussion of these next steps (Lines 433-436 in clean manuscript) in the discussion of the manuscript.

4. The study will benefit from employing a computational approach that can help in labeling calpain substrate cleavage sites from amino acid sequence using conditional random fields or any computational technique using the sequences of peptides obtained from the TAILS experiment.

We agree that this is a powerful technique, but until we have verification that the cleavage sites lost are directly due to loss of Calpain 15, this is premature. We have added the use of these computational approaches and our reasons for not using them at the present time (Lines 308-312 in clean manusript).

5. Since no substrate was validated and found in this study I suggest using other ages and not just P2 as well as focusing on a particular area of the brain such as cerebral cortex as opposed to using whole brain.

We agree that calpain 15 might be enriched in certain areas of the brain such as cerebral cortex and hippocampus. However, its expression in adult brain, in which we can isolate different brain areas to look for cleavage event as suggested, is minimal compared to prenatal and P2 brains.

6. I believe this current manuscript can be really strengthened by performing further in vivo validation experiments.

We agree that these would be important future steps, but they are beyond the scope of the present paper and would take additional years of research. We discuss these future steps in the discussion.

7. Figure 4: The images in figure 4 are not well aligned and seem scattered around.

We have revised this figure and done a better job with the alignment.

Additional Editor Comments:

While both reviewers commented favorably on your manuscript, some concerns need to be addressed. These include validation that the different sex of the mice did not affect the results and validation of more hits. The authors do not need to address the suggestion that other ages or specific regions of the brain should be investigated (concern #5 of one of the reviewers).

We have validated more hits and showed that our analysis already validated that the sex of the mice did not affect the results. We thank the editor for agreeing that adding more regions and more ages is outside the scope of this manuscript. We discuss that these will be important experiments for the future. Similarly, we have not done functional studies examining the effect of increases in the putative targest on neurodevelopment. Again, these will be important experiments for the future. We have included references for these targets showing that altering levels of these proteins has been shown to have effects in neurodevelopment in other studies.
