# Supplementary material for: Identifying putative substrates of Calpain-15 in neurodevelopment
Source: PLoS One. 2025 Apr 16;20(4):e0319489. doi: 10.1371/journal.pone.0319489 (PMC12002525; doi:10.1371/journal.pone.0319489)
Supplement: Attachment — Submitted filename: Response to Reviewers.pdf [file pone.0319489.s009.pdf]

Response to Reviewers

Dear Editor,

Thank you for the reviews. Below are the response to the reviewers. There were no comments from the academic editor. We hope the paper is now acceptable for publication at PLOS One.

Reviewer #1: All comments to the author has been addressed. I believe that the manuscript is technically sound and the data does support the conclusion.

Thank you for your support.

Reviewer #2: The authors have partly addressed comments as it seems more work would be needed to strengthen this study.

1. However, I think it's advisable for the authors to make the raw and processed RNA-seq data available by depositing in the GEO database. The proteomics data should also be deposited in a public repository database such as Global Proteome Machine Database (GPMDB), PeptideAtlas, the PRoteomics IDEntifications database (PRIDE), Tranche, and NCBI Peptidome.

We have deposited the RNA-SEQ data in the GEO database. We have also attached the full RNA-SEQ analysis as a supplemental data file. We have uploaded the proteomics data to the PRIDE database. The accession numbers have been added to the methods section.

If the reviewer wishes to examine these deposits, information is below. Data will be released to the public once we have the paper DOI information.

Reviewer access details

Log in to the PRIDE website using the following details:

Project accession: PXD060034

Token: qm4WU3lBbgV5

To review GEO accession GSE287980:
Go

to https://can01.safelinks.protection.outlook.com/?url=https%3A%2F%2Fwww.ncbi.nlm.nih.gov%2Fgeo%2Fquery%2Facc.cgi%3Facc%3DGSE287980&data=05%7C02%7Csenthil.duraikannukailasam%40mcgill.ca%7C65e4b9ef41ce4920189908dd3ca2fd8a%7Ccd31967152e74a68afa9fcf8f89f09ea%7C0%7C0%7C638733392919091956%7CUnknown%7CTWFpbGZsb3d8eyJFbXB0eU1hcGkiOnRydWUsIlYiOiIwLjAuMDAwMCIsIlAiOiJXaW4zMiIsIkFOIjoiTWFpbCIsIldUIjoyfQ%3D%3D%7C0%7C%7C%7C&sdata=I%2FkREJacdGS%2BBRt%2B3tnkLMLMwQENAkKQeRMrJF20zEA%3D&reserved=0
Enter token apwzwkmitbudtkz into the box

2. Additionally, the authors should discuss the strengths and limitations of the analysis from their study.

We had a section on the limitations of the study in the discussion, but it was not labeled. We have expanded this section and added strengths as well. This section of the discussion is reproduced below.

Strengths and Limitations of this study

This study has several strengths. It is the first examination of the effects of loss of Capn 15 on mRNA, protein levels, and cleavage products (TAILs) in any system and a number of significant changes were observed. Several of these were followed up by immunoblotting and significant changes in putative transcription factors that mediated the changes in RNA-SEQ were identified. This leads to strong hypotheses for future studies. There are limitations in this study. We focused on P2 brains as Capn15 is widely expressed at this time, but levels of Capn15 are higher in embryonic brain and many proteins whose loss is responsible for neurodevelopmental disorders act earlier during neuronal differentiation that peaks in embryonic brains. Capn15 may also be important in selected areas (such as the primordial eye fields) and using whole brains may dilute possible changes. It will be important in the future to determine if increases in the level of the proteins identified to be changed in Capn 15 Kos (Pax2, Pax5, Tubb3 and Dcx)

play a role in the phenotypes seen in this KO mouse. While the use of heterozygotes of wild type allowed for using littermates in the TAILS assay, it may have reduced the amount of changes in the affected peptides.
